# Efficacy of autologous platelet-rich plasma gel in the treatment of refractory pressure injuries and its effect on wound healing time and patient quality of life

**DOI:** 10.6061/clinics/2021/e2355

**Published:** 2021-02-01

**Authors:** Qian Liu, Ning Zhang, Zhengnan Li, Hongmei He

**Affiliations:** I Department of Sports Medicine, Ganzhou People's Hospital, Ganzhou, Jiangxi 341000, China; II Department of Gastroenterology, Ganzhou People's Hospital, Ganzhou, Jiangxi 341000, China

**Keywords:** Crush Injuries, Autologous Platelet-Rich Plasma Gel, Wound Healing, Quality of Life

## Abstract

**OBJECTIVES:**

To evaluate the efficacy of autologous platelet-rich plasma (PRP) gel in the treatment of refractory pressure injuries and its effect on wound healing time and quality of life of patients.

**METHODS:**

A random number table method was used to group 102 patients with refractory pressure injuries into either a control group (CG) (51 cases) receiving negative pressure wound therapy (NPWT) or a study group (SG) (51 cases) receiving NPWT+PRP gel.

**RESULTS:**

The total efficacy rate in the SG (92.16%) was higher than that in the CG (76.47%) (*p*<0.05). The SG exhibited lower visual analog scale (VAS) scores and pressure ulcer scale for healing (PUSH) scores, smaller wound sizes and depths, and shorter wound healing times than the CG after 21 days of treatment (*p*<0.05). After 6 months of treatment, the SG scored higher than the CG on the psychological, physiological, social functions, and daily activity domains on the World Health Organization Quality of Life (WHOQOL-BREF) scale (*p*<0.05). The incidence of postoperative complications in the SG (13.73%) was not significantly different from that of the CG (7.84%) (*p*>0.05).

**CONCLUSION:**

In the treatment of refractory pressure injuries, PRP gel can accelerate wound healing, reduce wound pain, shorten the treatment cycle, regulate tissue inhibitor matrix metalloproteinase-1 (TIMP-1) and matrix metalloproteinase-9 (MMP-9) levels and the expression of specific proteins in granulation tissue, reduce the levels of the inflammatory factors interleukin-1β (IL-1β), IL-8, and tumor necrosis factor-α (TNF-α), and improve the quality of life of patients without increasing complications.

## INTRODUCTION

Pressure injuries are localized damage to the skin and/or underlying tissue induced either by pressure alone or by a pressure-shear combination. These injuries often occur in patients on long-term bed rest, those with difficulty moving their lower extremities, and in patients with altered consciousness. Pressure injuries are associated with local tissue necrosis due to hypoxia/ischemia resulting from long-term compression (1,2). Refractory pressure injuries are mostly found in stage III and IV wounds. Stage III and IV pressure injuries are characterized by full-thickness tissue loss complicated by osteonecrosis and osteomyelitis, thereby increasing the risk of nutrient loss and infection, as well as impeding wound healing; furthermore, chronic sinuses form in these injuries due to the large amount of exudate from local wounds. These reasons make the prevention of refractory pressure injuries difficult (3,4). Routine treatments, such as antibiotic administration, dressings, and wound debridement are often needed; however, the treatment period is inherently long which inevitably increases the risk of wound infection. Negative pressure wound therapy (NPWT) can effectively promote wound healing, improve local blood flow, and reduce temporary swelling; however, NPWT materials are expensive which makes it difficult for some patients to avail (5). Platelet-rich plasma (PRP) gel is an emerging therapeutic option for chronic wounds; it is simple to manufacture and utilizes few consumables. This gel releases high concentrations of growth factors that stimulate cell proliferation and differentiation, seal the wound, accelerate hemostasis, repair damaged tissue, and promote regeneration or repair (6,7). However, there are currently few clinical reports on the use of autologous PRP gel dressings as adjuvant treatment for refractory pressure injuries. In view of this, this study explored the therapeutic effects and related mechanisms of autologous PRP gel dressing as an adjuvant therapy for refractory pressure injuries.

## MATERIAL AND METHODS

### Baseline data

This study enrolled 102 patients (52 men and 50 women) with refractory pressure injuries who were treated in our hospital between January 2018 and February 2020. The patients were aged 39-80 years, had a clinical course of 2-10 months, and were grouped according to the random number table method with 51 patients in both the control group (CG) and the study group (SG). This study was approved by the Ganzhou People's Hospital and complied with the relevant standards of the Declaration of Helsinki from the World Medical Association.

### Enrollment criteria

The inclusion criteria were as follows. The patient must 1) meet the diagnostic criteria for refractory pressure injuries according to the 2016 JDA Trauma/Burn Guidelines: Diagnosis and Treatment of Pressure Ulcers (8) for stages III-V wounds, 2) be older than 18 years of age, 3) not have diabetes, 4) voluntarily sign the informed consent forms, 5) maintain high treatment compliance, and 6) participate in the study until completion. The exclusion criteria were as follows. Patients who 1) were on hormone or immunosuppressant treatment up to 4 weeks before enrollment, 2) had existing hemorrhagic disease, malignant tumors, severe cardiovascular or cerebrovascular disease, severe malnutrition, autoimmune disease, or mental illness, and 3) had any form of coagulopathy.

## METHODS

The surface of the wound was wiped with 0.9% sodium chloride. Debris and necrotic tissues were removed with tweezers, forceps, or scissors, and the wound surface was then dried using gauze dressings. In patients with osteomyelitis, necrotic bone tissue was completely cleared. Afterwards, the CG received NPWT treatment. Depending on the shape and size of the wound, NPWT material (consumable material for the negative pressure-assisted healing treatment system [KCI USS, V.A.C. black auxiliary material]) was trimmed and used to cover the wound. Both the wound surface and a porous drainage tube were bandaged using foam materials and sealed with transparent films. A negative pressure drainage device (Wuhan VSD Medical Science & Technology Co., Ltd.) was connected to the drainage tube and placed on either a continuous or intermittent negative pressure suction mode (suction pressure setting from to 450-600 mmHg [1 mmHg≅0.133 kPa]). The negative pressure suction material was replaced as appropriate. In addition to the treatment described above, the SG was also treated with PRP gel. Autologous PRP gel was prepared in a clinical laboratory by a professional physician. A total of 10-20 mL of peripheral venous blood was collected from the patient and centrifuged for 10 min (1,500 rpm, 6 cm) to discard the lower red blood cell layer. After re-suspension and further centrifugation for 10 min (1500 rpm, 6 cm), the supernatant was discarded to obtain the middle layer. This was then mixed with calcium and thrombin and subsequently coagulated to create PRP gel. This was evenly and quickly sprayed to the wound surface and sealed with sterile dressing. Treatment was repeated every 7 days for a total of three times.

### Evaluation index

1. Curative effect: The outcome was defined as a “cure” if, after 21 days of treatment, the wound surface was completely covered by epithelium and fresh granulation tissue had formed. The outcome was defined as an “improvement” if purulent secretions from the wound were reduced, the wound size was reduced by more than 25%, and if fresh granulation tissue appeared. If purulent secretions from the wound were not significantly reduced and the wound expanded or had ≤25% reduction in size, the treatment was defined as “invalid”. The effective rate was calculated as “(improvement+cure)/total number of cases”.
2. Indicators of wound healing: The wound healing time, visual analog scale (VAS) scores, and pressure ulcer scale for healing (PUSH) scores were recorded before and after 21 days of treatment. The VAS score ranges from 0 to 10 points; a higher score indicates increased pain severity. The PUSH score assesses changes in pressure injury status over time and includes the wound size, the amount of exudates, and the tissue type in its computation with a total score of 17 points; higher scores denote a poorly healed wound.
3. The size and depth of the wound surface: The size and depth of wounds were recorded before and after 21 days of treatment. For regular wound surfaces, the maximum diameters of the length and width of the wound were measured with a ruler for calculation. For irregular wounds, different lengths and widths were measured for calculation. To measure the depth of the wound, a probe was inserted into the bottom of the wound and the portion that is level with the epidermis was noted; the depth was then obtained by measuring the length from the end of the probe to the level of the epidermis with a ruler.
4. Granulation tissue-specific protein expression: Before treatment and after 21 days of treatment, the granulation tissue cells of each patient's wound were lysed and centrifuged to extract the total protein contents. After electrophoresis and membrane transfer, the levels vascular endothelial growth factor (VEGF), stromal cell-derived factor-1α (SDF-1α), and chemokine receptor 4 (CXCR4) were determined.
5. Serum indicators: 3 mL of fasting venous blood was collected before and after 21 days of treatment. The samples were centrifuged at 3000 rpm for 10 min. Interleukin-1β (IL-1β), IL-8, tumor necrosis factor-α (TNF-α), tissue inhibitor matrix metalloproteinase-1 (TIMP-1), and matrix metalloproteinase-9 (MMP-9) levels were measured by enzyme-linked immunosorbent assays purchased from Beijing Meikang Gene Science Co., Ltd., Beijing, China.
6. Quality of life: The World Health Organization Quality of Life Instruments (WHOQOL-BREF) was used to assess patients’ quality of life before and after 6 months of treatment in terms of psychological, physiological, and social functions, as well as daily activities, with a score of 0-100 points in each dimension. Higher scores indicate a higher quality of life (9).
7. Complications: Symptoms of infection, allergy, rash, fluid accumulation, redness, or swelling during treatment were recorded.

All indicators were evaluated by the same attending physician.

### Statistical Analysis

Data processing was performed through IBM Statistical Package for the Social Sciences (SPSS) version v22.0 (IBM Corp, NY, USA) for Windows. Measurement data (mean±SD) were compared using a t-test, and count data were expressed as a percentage and tested using the chi-square test. A *p*-value <0.05 was considered statistically significant.

## RESULTS

### Comparison of baseline data

All 102 patients with refractory pressure injuries met the inclusion and exclusion criteria and no cases were excluded. No significant differences in sex, age, course of disease, pressure injury staging, and location of the pressure injury were identified between the two groups (*p*>0.05) (Table 1).

### Comparison of clinical efficacy

The total efficacy rate was significantly higher in the SG (92.16%) than in the CG (76.47%) (*p*<0.05) (Table 2).

### Comparison of wound healing-related indices

**Figure 1:**
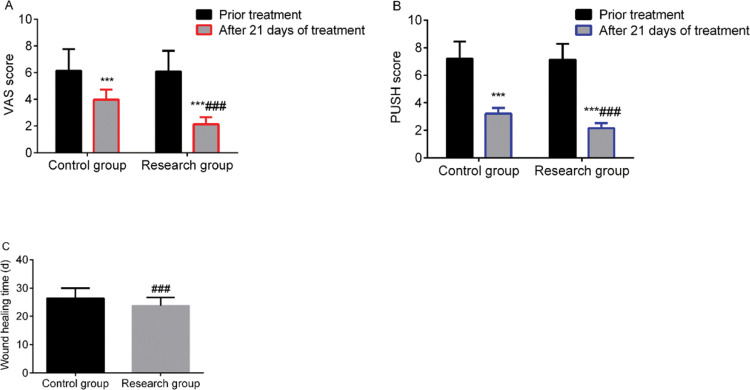
Comparison of wound healing indices between the two groups. A: VAS score, B: PUSH score, C: wound healing time. *** indicates *p*<0.001 when compared to levels before treatment; ^###^ indicates *p*<0.001 when compared to the control group. VAS: Visual analog scale; PUSH: Pressure Ulcer Scale for Healing.

Before treatment, VAS scores, PUSH scores, and wound healing times did not differ between the two groups (*p*>0.05). However, after 21 days of treatment, VAS and PUSH scores were significantly lower, and wound healing time was significantly shorter, in the SG than in the CG (*p*<0.05) (Figure 1).

### Comparison of wound size and depth

**Figure 2:**
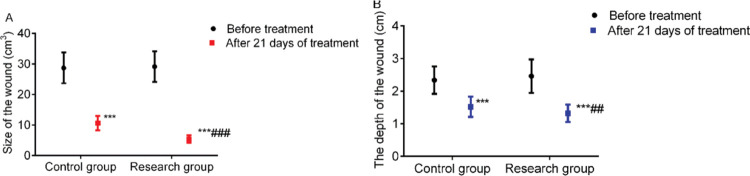
Comparison of wound size and depth between the two groups. A: Wound size, B: Wound depth. *** indicates *p*<0.001 when compared to levels before treatment; ^###^ indicates *p*<0.001 when compared to the control group.

Before treatment, there was no significant difference in the size and depth of the wounds between the two groups (*p*>0.05). After 21 days of treatment, these parameters were significantly reduced in the SG compared to those in the CG (*p*<0.05) (Figure 2).

### Comparison of granulation tissue-specific protein expression

**Figure 3:**
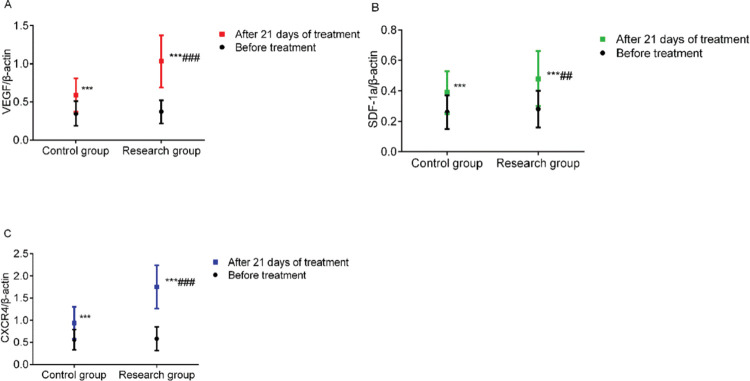
Comparison of granulation tissue-specific protein expression. A: VEGF levels, B: SDF-1α levels, and C: CXCR4 levels. *** indicates *p*<0.001 when compared to levels before treatment; ^###^ indicates *p*<0.001 when compared to the control group. VEGF, vascular endothelial growth factor; SDF-1a, stromal cell-derived factor-1α; CXCR4, chemokine receptor 4.

Before treatment, the levels of VEGF, SDF-1α, and CXCR4 were compared, and no significant differences were found between the two groups (*p*>0.05). After 21 days of treatment, the levels of VEGF, SDF-1α, and CXCR4 levels in the SG were higher than those in the CG (*p*<0.05) (Figure 3).

### Comparison of the levels of inflammatory markers

**Figure 4:**
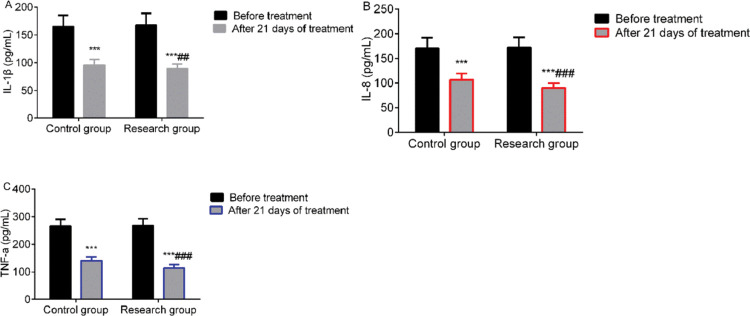
Comparison of the levels of inflammatory markers. A: IL-1β levels; B: IL-8 levels; C: TNF-α levels. *** indicates *p*<0.001 when compared to levels before treatment; ^###^ indicates *p*<0.001 when compared to the control group. IL-1β: Interleukin-1β; TNF-α: Tumor necrosis factor-α.

Before treatment, the levels of IL-1β, IL-8, and TNF-α were not significantly different between the two groups (*p*>0.05). After 21 days of treatment, the levels in the SG were lower than those in the CG (*p*<0.05) (Figure 4).

### Comparison of TIMP-1 and MMP-9 levels

**Figure 5:**
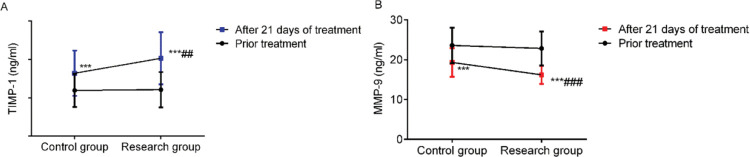
Comparison of TIMP-1 and MMP-9 levels. A: TIMP-1 levels and B: MMP-9 levels. *** indicates *p*<0.001 when compared to levels before treatment; ^###^ indicates *p*<0.001 when compared to the control group. TIMP-1: matrix metalloproteinase inhibitor-1; MMP-9: matrix metalloproteinase-9.

Before treatment, there was no significant difference in the levels of TIMP-1 and MMP-9 between the two groups (*p*>0.05). After 21 days of treatment, the SG showed higher levels of TIMP-1 and lower levels of MMP-9 than the CG (*p*<0.05) (Figure 5).

### Comparison of quality of life

Before treatment, the psychological, physiological, and social functions, as well as the daily activity scores of the WHOQOL-BREF scale were compared, and there were no statistically significant differences between the two groups (*p*>0.05). Six months after treatment, the scores were significantly increased in both the CG and SG, but the SG exhibited higher scores in all dimensions *(p<*0.05) (Table 3).

### Comparison of the incidence of complications

There was no significant difference in the incidence of postoperative complications between the SG (13.73%) and the CG (7.84%) (*p*>0.05) (Table 4).

## DISCUSSION

Refractory pressure injuries are mainly characterized by persistent symptoms and severe wound infections; these characteristics are related to blocked microcirculation, reduced local blood flow, attenuated phagocytosis, accelerated destruction of tissue proteins, and wound infection. Therefore, improving cell migration in scaffolds, activating cell proliferative activity, and reducing wound inflammatory responses are key parameters involved in the acceleration of wound repair (10,11). NPWT is a common method to treat refractory pressure injuries and has the following advantages: it insulates the wound and avoids secondary infection; promotes the exudation of wounded and necrotic tissue from the body, and increases blood flow in the wound and accelerates the growth of granulation tissue through negative pressure stimulation. However, NPWT materials are more expensive and can only be used as a transitional means.

In this study, when compared to the CG, the SG, which was treated with PRP, had a higher effective rate and higher WHOQOL-BREF scale scores, while also having lower VAS and PUSH scores, smaller wound size, and more shallow wound depths after 21 days of treatment. Fu et al. found that the total effective rate of autologous PRP gel combined with NPWT for the treatment of diabetic foot ulcers was 95.74% (12), which was significantly higher than that of conventional treatments and is consistent with the results of this study. It can be seen that, in the context of treatment with NPWT, the use of autologous PRP gel had obvious effects, including accelerated wound healing, reduced wound pain and size, and improved patient quality of life. The underlying reasons behind these effects may be related to several mechanisms of action of PRP gels. Autologous PRP contains fibrin, anti-platelets, cytokines, and growth factors which, after gel formation, can create an environment with low oxygen and humidity, accelerate the secretion of growth factors and cytokines, promote cell proliferation in wounds, and regulate the degradation and synthesis of the extracellular matrix (13). Autologous PRP also promotes vascular regeneration, fibroblast proliferation, and local blood flow reconstruction, provides supplementary growth factors to wounds, and exerts antimicrobial effects by promoting the proliferation of vascular endothelial cells and fibroblasts, inducing the activation, chemotaxis, and phagocytosis of monocytes and neutrophils, and accelerating the formation of granulation tissue (14,15). The preparation of PRP gels includes the use of a fully automated plasma separation and replacement method and density-gradient centrifugation. These operations are simple, convenient, safe, and have little risk for immune rejection. In this study, there was no significant difference in the incidence of complications between the two groups. Guoyou et al. (16) applied PRP gel on 12 patients with stage-IV pressure injuries, and all patients grew fresh granulation tissue after two dressing changes. No obvious adverse reactions or complications were observed during treatment. Therefore, this study further confirmed the safety of PRP gels.

Clinical studies have revealed that wound healing is comprised of three main phases: inflammation, granulation, and tissue reconstruction. Furthermore, it is known that maintaining the dynamic balance of the extracellular matrix (ECM) plays a vital role in the process of wound healing (17). Matrix metalloproteinases and TIMPs can affect the decomposition and synthesis of ECM, and MMP/TIMP systems are closely related to numerous cytokines and growth factors. These are important enzyme systems for remodeling and degrading the extracellular matrix, and the expression and timed release of MMPs/TIMPs can affect the wound healing process (18). In this study, the levels of TIMP-1 in the SG after 21 days of treatment were higher, and the levels of MMP-9 were lower than that of the CG, indicating that PRP gel can regulate the levels of TIMP-1 and MMP-9 and accelerate wound healing.

During the inflammatory reaction phase, vascular permeability is increased, macrophages, monocytes, and neutrophils are activated, and the release of many growth factors, chemokines, and cytokines is promoted (19,20). Among these, pro-inflammatory factors, such as IL-1β and TNF-α, can further increase tissue inflammation. Tumor necrosis factor-α can increase the level of MMPs, induce macrophages to produce IL-1β, and affect the formation of collagen on wound surfaces. Excessive inflammation will hinder wound healing (21). After 21 days of treatment, IL-1β, IL-8, and TNF-α levels in the SG were lower than those in the CG, and the levels of VEGF, SDF-1α, and CXCR4 in the SG were higher than those in the CG. This shows that PRP gel can reduce inflammation levels, regulate the expression of granulation tissue-specific proteins, and inhibit the progression of the disease. The reason behind this effect may be due to the high concentration of growth factors in PRP gels which circumvents the insufficient effects of single growth factors in repairing damaged tissues and promoting cell division. Furthermore, these growth factors promote adhesion to tissue defects, prevent platelet loss, and provide the most favorable environment for tissue repair and cell regeneration (22,23). In addition, the growth factors and fibrin contained in PRP gels can inhibit or eliminate the release of inflammatory cells from the wound surface, increase the levels of MMP-9 and the rate of angiogenesis, and promote the growth of granulation tissue. The expression of specific proteins in granulation tissue can reflect the growth of granulation tissue itself. Stromal cell-derived factor-1α belongs to the chemokine protein family and is a small cytokine that plays an important role in development. Chemokine receptor type 4 is a specific receptor of CXCL12, which has a strong chemotactic effect on lymphocytes. VEGF contained in the PRP gel can promote the invasion of microvascular endothelial cells into collagen gels and induce bone formation by regulating the expression of specific proteins, such as SDF-1α and CXCR4, thereby promoting cell proliferation and differentiation, accelerating neovascularization, regulating the immune response, and promoting the growth of granulation tissue (24,25).

In summary, PRP gel had a definite effect that could accelerate wound healing, reduce wound pain, shorten the treatment cycle, reduce inflammation, regulate TIMP-1 and MMP-9 levels, regulate specific protein expression in granulation tissue, and improve patient quality of life without increasing complications. However, due to the small and single sample size of this study, the results may be biased to some extent. At the same time, PRP gel was mainly administered in this study by spraying, but we also found that the injection of autogenous PRP gel at the site of injury is very effective in clinical practice. Due to the limitations of funding and the small number of cases, the difference in efficacy between the two was not studied. In the future, the number of samples can be increased to provide an in-depth discussion.

## AUTHOR CONTRIBUTIONS

Liu Q and He H conceived the study and designed the experiments. Zhang N and Li Z contributed to data collection, performed the data analysis, and interpreted the results. Liu Q wrote the manuscript. He H contributed to the critical revision of the article. All autho rs have read and approved the final version of the manuscript.

## Figures and Tables

**Table 1:** Comparison of baseline data.

				Pressure injury staging	Location of pressure injuries
Group	Male/Female	Age (years)	Course of pressure injuries (months)	Phase II/Phase III/Phase IV	Buttocks/Sacrococcygeal region/Greater trochanter
Control group (n=51)	27/24	59.36±6.21	6.21±2.12	15/24/12	28/20/3
Study group (n=51)	25/26	60.79±6.38	6.34±2.34	13/25/13	30/17/4
t/χ^2^/Z	0.157	1.147	0.294	0.413	0.203
*p*	0.692	0.254	0.769	0.680	0.903

**Table 2:** Comparison of treatment efficacy between the two groups (n (%)).

Group	Cure	Improvement	Invalid	Total effective rate
Control group (n=51)	16 (31.37)	23 (45.10)	12 (23.53)	39 (76.47)
Study group (n=51)	21 (41.18)	26 (50.98)	4 (7.84)	47 (92.16)
χ^2^				4.744
*p*				0.029

**Table 3:** Comparison of WHOQOL-BREF scale scores (Mean scores±S).

	Psychological function		Physiological function		Social function		Daily activities	
Group	Before treatment	After 6 months of treatment	Before treatment	After 6 months of treatment	Before treatment	After 6 months of treatment	Before treatment	After 6 months of treatment
Control group (n=51)	57.76±8.67	69.26±11.24***	59.34±9.37	70.26±10.49***	61.51±10.65	72.36±12.26***	54.32±9.16	66.37±13.34***
Study group (n=51)	58.86±9.31	76.37±12.27***	60.08±10.05	78.85±11.37***	62.37±11.29	81.36±13.37***	56.29±10.87	73.64±14.29***
t	0.617	3.051	0.385	3.965	0.396	3.543	0.990	2.656
*p*	0.539	0.003	0.701	0.000	0.693	0.001	0.325	0.009

*** indicates *p*<0.001 compared with the scores before treatment within the same group.

**Table 4:** Comparison of postoperative complications between the two groups (n (%)).

Group	Infection	Allergies & rashes	Effusion	Redness	Total
Control group (n=51)	1 (1.96)	0	2 (3.92)	1 (1.96)	4 (7.84)
Study group (n=51)	1 (1.96)	3 (5.88)	2 (3.92)	1 (1.96)	7 (13.73)
χ^2^					0.917
*p*					0.338
